# Adhesive and injectable hydrogel microspheres for NRF2-mediated periodontal bone regeneration

**DOI:** 10.1038/s41368-024-00340-w

**Published:** 2025-01-10

**Authors:** Yu Wang, Shanshan Jin, Yaru Guo, Yilong Lu, Xuliang Deng

**Affiliations:** 1 Department of Orthodontics, Peking University School and Hospital of Stomatology & National Center for Stomatology & National Clinical Research Center for Oral Diseases & National Engineering Laboratory for Digital and Material Technology of Stomatology, Beijing, China; 2 Department of Pediatric Dentistry, Peking University School and Hospital of Stomatology & National Center for Stomatology & National Clinical Research Center for Oral Diseases & National Engineering Laboratory for Digital and Material Technology of Stomatology, Beijing, China; 3 Department of Geriatric Dentistry, Peking University School and Hospital of Stomatology & National Center of Stomatology & National Clinical Research Center for Oral Diseases & National Engineering Laboratory for Digital and Material Technology of Stomatology & Beijing Key Laboratory of Digital Stomatology & Research Center of Engineering and Technology for Computerized Dentistry Ministry of Health & NMPA Key Laboratory for Dental Materials, Beijing, China

**Keywords:** Mesenchymal stem cells, Regenerative medicine

## Abstract

Regenerating periodontal bone defect surrounding periodontal tissue is crucial for orthodontic or dental implant treatment. The declined osteogenic ability of periodontal ligament stem cells (PDLSCs) induced by inflammation stimulus contributes to reduced capacity to regenerate periodontal bone, which brings about a huge challenge for treating periodontitis. Here, inspired by the adhesive property of mussels, we have created adhesive and mineralized hydrogel microspheres loaded with traditional compound cordycepin (MMS-CY). MMS-CY could adhere to the surface of alveolar bone, then promote the migration capacity of PDLSCs and thus recruit them to inflammatory periodontal tissues. Furthermore, MMS-CY rescued the impaired osteogenesis and ligament-forming capacity of PDLSCs, which were suppressed by the inflammation stimulus. Moreover, MMS-CY also displayed the excellent inhibitory effect on the osteoclastic activity. Mechanistically, MMS-CY inhibited the premature senescence induced by the inflammation stimulus through the nuclear factor erythroid 2-related factor (NRF2) pathway and reducing the DNA injury. Utilizing in vivo rat periodontitis model, MMS-CY was demonstrated to enhance the periodontal bone regeneration by improving osteogenesis and inhibiting the osteoclastic activity. Altogether, our study indicated that the multi-pronged approach is promising to promote the periodontal bone regeneration in periodontitis condition by reducing the inflammation-induced stem cell senescence and maintaining bone homeostasis.

## Introduction

Periodontitis is regarded as one of the most popular inflammatory diseases. It is typical of the biofilm-induced inflammatory damage, causing the disordered periodontal ligament and lost alveolar bone, which further bring abouts tooth loosening and even tooth loss.^1^ Traditional mechanical therapy or the local usage of antibiotics and antimicrobials for periodontitis aim to improve the periodontal microbiota symbiosis or control the inflammation. However, these methods are incapable of actively recovering the impaired function of periodontal tissue and fully motivating the endogenous regenerative potential.^2^ Besides, traditional periodontal surgery, such as the guided tissue regeneration, still faces huge uncertainty in regenerating the lost bone, and the general outcomes possess a high clinical unpredictability.^3,4^ Therefore, periodontal bone regeneration remains a considerable challenge for clinicians and scientists.

Periodontal regeneration mainly relys on the osteogenic capacity of PDLSCs, which belong to a specific population of mesenchymal stem cells that inhabited in the periodontal ligament. During the development and progress of periodontitis, their osteogenic ability is hampered due to chronic inflammation, accompanied by reduced proliferative and migratory capacity and eventually hinders periodontal regeneration.^5^ Recently, many studies have reported that the chronic inflammatory stimulus could result in the occurrence of the premature senescence, which becomes a significant hurdle for bone regeneration and repair.^6^ Exploring efficient therapeutic strategies for inhibiting inflammation-induced senescence of stem cells represent a promising choice for boosting bone repair and regeneration. NRF2 is associated with the premature senescence of stem/progenitor cells and contributes to tissue dysfunction in humans and mice.^7^ According to previous reports, the activation of the KEAP1-NRF2 signaling system could protect cells from the stress damage and delay the cellular senescence.^8^ More importantly, NRF2 activation ultimately increases the mRNA expression of several antioxidant genes, such as NQO1 and HO-1, which are responsible for the recovery of impaired cell function.^9^ Thus, a targeted strategy for activating NRF2 in PDLSCs to suppress the inflammation-induced premature senescence is crucial for recovering their reduced osteogenic capacity, thus enhancing the periodontal bone regeneration.

Cordycepin (3′-deoxyadenosine) belongs to one of the main bioactive components of Cordyceps militaris that derived from the nucleoside adenosine.^10^ Previous studies have revealed that it exerted some therapeutic properties, such as anti-inflammation, anti-aging, and anti-tumor.^11^ Its comprehensive pharmacological function suggests that cordycepin possess great promise in clinical application. Interestingly, many studies have demonstrated that it was able to delay the osteoporosis by inhibiting the osteoclastic activity, which indicated that it may hold immense potential in treating the osteoclastogenesis-related periodontitis.^12^ However, high concentrations of cordycepin slow down the cell cycle and produce liver and kidney toxicity.^13^ Therefore, sustained cordycepin delivery systems are needed to realize its controlled release and reduce its’ adverse reactions.

Because of the high-water content, soft structure, and high porosity, different kinds of hydrogel-based drug delivery tools have recently been attempted, including thermosensitive, photosensitive, pH-sensitive, as well as active targeting hydrogels.^14^ Due to the remarkable differences between the characteristics of various tissues, tissue-adaptive hydrogel systems should be considered to optimize their effects. The periodontium includes the periodontal ligament, gingiva and alveolar bone, which forms a relatively closed tissue microenvironment for nutrient exchange and cell communication.^15^ The alveolar bone is covered by the gingiva, which prevents ultraviolet light from penetrating the gingiva to realize the cross-linking of hydrogel.^16^ Moreover, the simple injection of therapeutic drugs is unable to accumulate in the diseased areas and maintain the efficient therapeutic concentration in vivo. Therefore, hydrogels with injectable capacity, adhesive ability and porous structure, are ideal equipments to serve as the drug carrier in periodontal tissues. Hydrogel microspheres are more convenient to be locally injected in non-invasive manners and can be produced by microfluidics, electro-hydrodynamic spraying and batch emulsion. Mussel-inspired adhesive modification has been applied in various studies.^17^ By modifying the surface of the multiple kinds of scaffolds with polydopamine (PDA) solution, the adhesive capacity of biomaterials can be relatively improved. Therefore, this technology may also improve the microsphere adhesion on the alveolar bone.^18^ To further accelerate the bone formation, osteoinductive calcium phosphates are often adopted. The PDA coating could increase nucleation sites for apatite deposition, which eventually realized the forming of biomineralization layers.^19^ Therefore, in thought of the adhesive property of PDA coating and the features of periodontium anatomy, adhesive and mineralized hydrogel microspheres were designed for realizing localized cordycepin delivery.

In our study, methacrylonitrile acylation gelatin microspheres produced by batch emulsion were modified by polydopamine on their surface and biomineralized by simulated body fluids and calcium ion solution, eventually prepared the adhesive and mineralized hydrogel microsphere that could be injected and adheres to the local areas and delivers the PLGA-loaded cordycepin (MMS-CY). This system could adhere to the wet surface of alveolar bone and possess controlled drug releasing property. In vitro experiments demonstrated that MMS-CY could promote PDLSCs’ migratory, osteogenic and ligament-forming capacity, and inhibit their excessive osteoclast activity. Mechanistically, MMS-CY could decrease the secretion of inflammatory factors and reduce DNA injury by activating the NRF2 pathway in PDLSCs. In vivo animal experiments demonstrated that MMS-CY could promote the periodontal bone regeneration in periodontitis condition. Overall, our study provides a promising strategy for improving stem cell function and reducing the inflammation-induced stem cell senescence to promote the repair and regeneration of bone defect in periodontitis condition.

## Results

### Preparation and characterization of MMS-CY

**Fig. 1:**
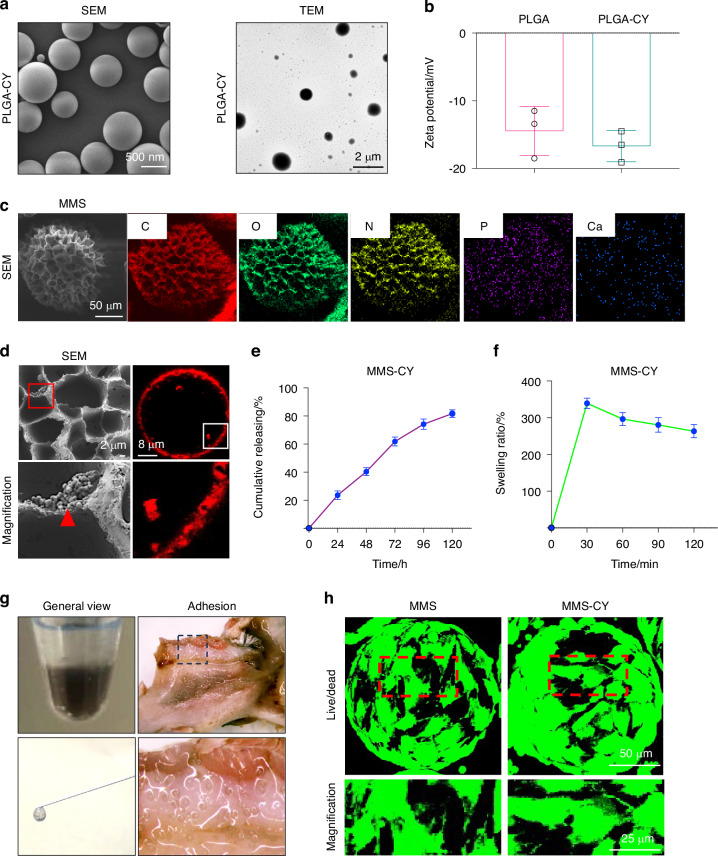
Characterization of MMS-CY. **a** SEM and TEM images of PLGA-CY. **b** Zeta potential of PLGA and PLGA-CY. **c** Elemental mapping images of MMS-CY. **d** SEM images and immunofluorescence images of MMS-CY. **e** Release profiles of cordycepin from MMS-CY. **f** Swelling ratio of MMS-CY. **g** Overall views of injectable MMS-CY. **h** Live/dead assay staining of PDLSCs cultured on different kinds of microspheres

In our study, biodegradable poly biodegradable poly (d, l-lactide-co-glycolide) (PLGA)- nanoparticles were used to load the drug cordycepin through an oil/water (O/W) emulsion-solvent evaporation method. Besides the images from scanning electron microscopy (SEM), the transmission electron microscopy (TEM) images also revealed that the CY-nps were uniform round spheres, which displayed an average diameter that mainly ranged from 300 nm to 600 nm (Figs. 1a and S1). Their surface zeta potential was −16.7 mV because of the negatively charged with carboxyl on their surface (Fig. 1b). The batch emulsion method was adopted to prepare the monodisperse GelMA microspheres. The porous structure was realized through several rounds of freeze-drying process, which was based on phase separation. Then, the polydopamine modification was achieved by immersing the microspheres in the dopamine aqueous solution. Finally, the PDA-modified microspheres were immersed into 5-times SBF (5SBF) and CaCl_2_ solution to finish the process of apatite deposition. From Fig. 1c, the calcium and phosphorous element mapping demonstrated the realization of the biomineralization. The scanning electron microscope results also showed that the mineralization and PDA-coating maintain the their porous microstructure, providing a suitable space for drug-loaded nanoparticles to absorb fully. Because mineralized microspheres (MMSs) had a three dimensional network structure, which make them absorb enough fluids. Therefore, the microspheres were immersed in the CY-nps with stirring at 4 °C to load the drugs. SEM images showed that CY-nps were distributed evenly on the surface of the microspheres (Fig. 1d). To further demonstrate the efficient loading of CY-nps on the microspheres, the fluorescence microscope was utilized to examine the Dil-labeled PLGA nanoparticles. Immunofluorescence staining shown that the edges of microspheres were distributed with red staining signaling, indicating the efficient loading of CY-nps (Fig. 1d). The subsequent drug release experiments showed that the MMS-CY exhibited a fast release at the initial stage, which then followed by a slower release feature (120 h) (Fig. 1e), indicating that the MMS-CY can act as an efficient drug delivery tools. Next, we examined the swelling property of MMS-CY. Results showed that MMS-CY swelled to 300% within 0.5 h, decreased slowly, and finally maintain the level of 293% after 120 mins (Fig. 1f).

It is vital for realizing the local delivery at the periodontitis site that the drug system possess outstanding injectability. As shown in Fig. 1g, our MMS-CY displays excellent injectability. Moreover, owing to the PDA coating, the MMS-CY can adhere to the surface of the surrounding tissues of the mandibular first molar in wet conditions. As a drug delivery base, MMS-CY also allows the cells to adhere to their surface. The live/dead assay showed that PDLSCs in MMS and MMS-CY groups distributed on the surface and displayed good viability (Fig. 1h). Collectively, these results indicated that MMS-CY possesses excellent drug-loading capacity and waterproof adhesive properties.

### MMS-CY possessed excellent biocompatibility and enhanced PLDSCs’ migratory capacity

**Fig. 2:**
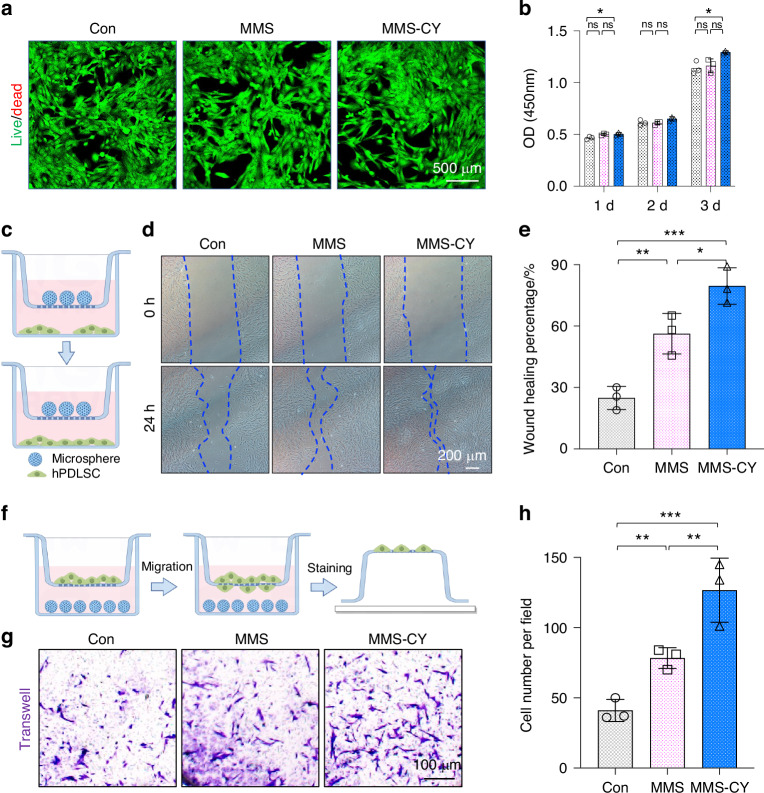
Assessment of MMS-CY biocompatibility and recruitment capacity. **a** Live/dead assay of MMS and MMS-CY-treated PDLSCs. **b** CCK-8 test of MMS and MMS-CY-treated PDLSCs (*n* = 3 biologically independent samples, by one-way ANOVA Turkey’s multiple comparison test: ns: not significant, *P < 0.05). **c** Schematic diagram of the scratch wound healing assay. **d** Microscope images of scratch lines at 0 and 24 h. **e** Semi-quantification of the percentage of wound healing (*n* = 3 biologically independent samples, by one-way ANOVA Turkey’s multiple comparison test: ***P < 0.001, **P < 0.01, *P < 0.05). **f** Schematic diagram of the chemotaxis experiment. **g** Microscope images of the migration of PDLSCs from the upper chamber. **h** Semi-quantification of the number of migrating cells. (*n* = 3 biologically independent samples, by one-way ANOVA Turkey’s multiple comparison test: ***P < 0.001, **P < 0.01). Data are represented as mean ± SD

PDLSCs are indispensable for preserving the periodontal bone homeostasis and promoting bone regeneration.^20^ Therefore, we chose PDLSCs as a in vitro cell model to assess the biological function of MMS-CY on stem cells. We made use of a coculture system for MMS-CY and PDLSCs by use of the transwell and assessed the effect of MMS-CY on the PDLSCs’ viability and their proliferation according to the live/dead assay and CCK-8 experiment. As shown in Fig. 2a, b, nearly all PDLSCs remained viable after the 3-day culture in different groups. Consistently, results from the CCK-8 experiments showed that the PDLSCs from different groups keep the increasing trend. Importantly, MMS-CY displays the highest proliferative ability.

To simulate the mode of action of MMS-CY on PDLSCs in vivo, the specific culture system for MMS-CY and PDLSCs was constructed by transwell apparatus. In the scratch wound healing assay, PDLSCs were cultured in the lower chambers. Then, the MMS-CY was given into the upper chambers. Results showed that the percentage of wound healing in the MMS-CY group was highest compared with the MMS and control group (Fig. 2c–e), indicating that MMS-CY improved the migratory ability of PDLSCs through releasing cordycepin. As for the chemotaxis assay, PDLSCs were added onto the upper chamber while the lower chamber was supplemented with different kinds of microspheres. As shown in Fig. 2f–h, the number of migrated cells was remarkably increased after the MMS-CY supplementation, indicating that MMS-CY may mobilize PDLSCs to migrate to the destination site. Overall, the MMS-CY improved the migratory ability of PDLSCs and enhanced their attachment. Since recruitment and migration of PDLSCs are crucial for the periodontal regeneration, MMS-CY might be thus used to promote the periodontal regeneration.

### MMS-CY rescued impaired osteogenic differentiation capacity of PDLSCs under inflammation stimulation

**Fig. 3:**
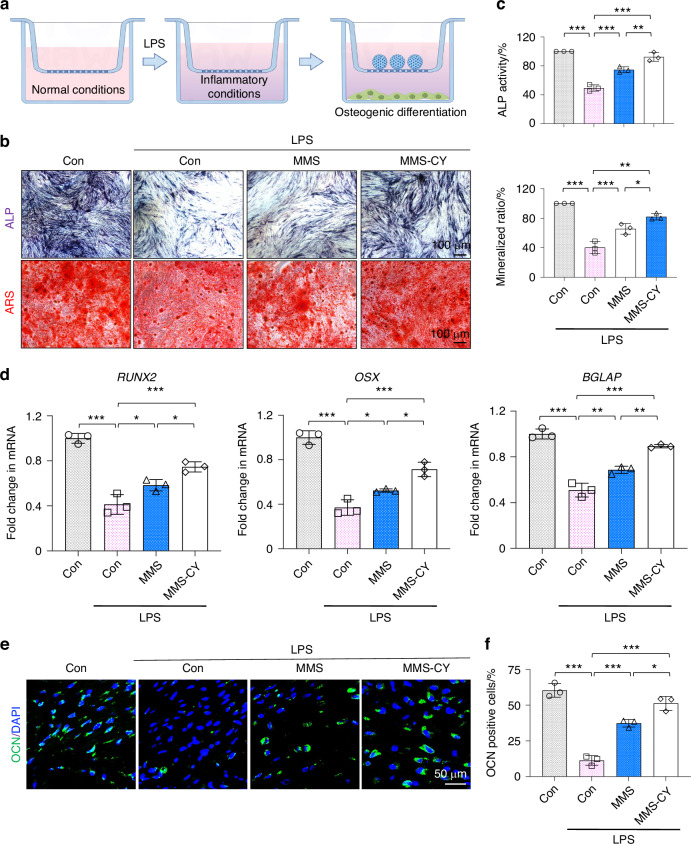
MMS-CY improves the osteogenic capacity of PDLSCs under the inflammation stimulus. **a** Schematic diagram of MMS-CY restoring the impaired osteogenic differentiation capacity of PDLSCs under the inflammatory condition. **b** ALP and ARS staining of PDLSCs after coculture with MMS and MMS-CY for 7 days and 14 days respectively. **c** Quantification of the ALP activity on day 7 and the ratio of ARS positive areas in total areas on day 14 (*n* = 3 biologically independent samples, by one-way ANOVA Turkey’s multiple comparison test: ns: not significant, ***P < 0.001, **P < 0.01, *P < 0.05). **d** RT-qPCR of the osteogenic gene expression RUNX2, OSX and BGALP (*n* = 3 biologically independent samples, by one-way ANOVA Turkey’s multiple comparison test: ***P < 0.001, **P < 0.01, *P < 0.05). **e** Immunofluorescence staining of OCN of MMS and MMS-CY-treated PDLSCs under the inflammatory condition. **f** Semi-quantification of OCN (*n* = 3 biologically independent samples, by one-way ANOVA Turkey’s multiple comparison test: ***P < 0.001, *P < 0.05). Data are represented as mean ± SD

Under periodontitis condition, the inflammation stimulus could suppress the osteogenic ability of PDLSCs.^21^ Therefore, we explored the therapeutic effect of MMS-CY on the impaired osteogenic capacity (Fig. 3a). LPS (Lipopolysaccharide) was used to construct the inflammatory environment. ALP and ARS results revealed that the LPS stimulus remarkably inhibited the ALP activity and reduced the formation of mineralized nodules. Similarly, the RT-qPCR results also demonstrated that the transcriptional expression of osteogenic genes, including RUNX2, Osterix (OSX) and Osteocalcin (OCN) were significantly downregulated in LPS-stimulated PDLSCs. By contrast, the application of MMS-CY remarkably increased the osteogenic activity compared with the MMS group under the inflammatory condition, as evidenced by higher levels of ALP activity, the increased amounts of calcium nodules and the elevated mRNA expression (Fig. 3b–d). Furthermore, the immunofluorescence staining showed that the application of MMS-CY increased the percentage of OCN positive cells under the inflammatory condition (Fig. 3e, f). Together, the above results demonstrated that MMS-CY could improve the impaired osteogenic capacity induced by the inflammatory stimulus, indicating that MMS-CY has excellent potentials for the periodontal bone regeneration.

### MMS-CY rescued the impaired ligament-forming capacity under the inflammation stimulus

**Fig. 4:**
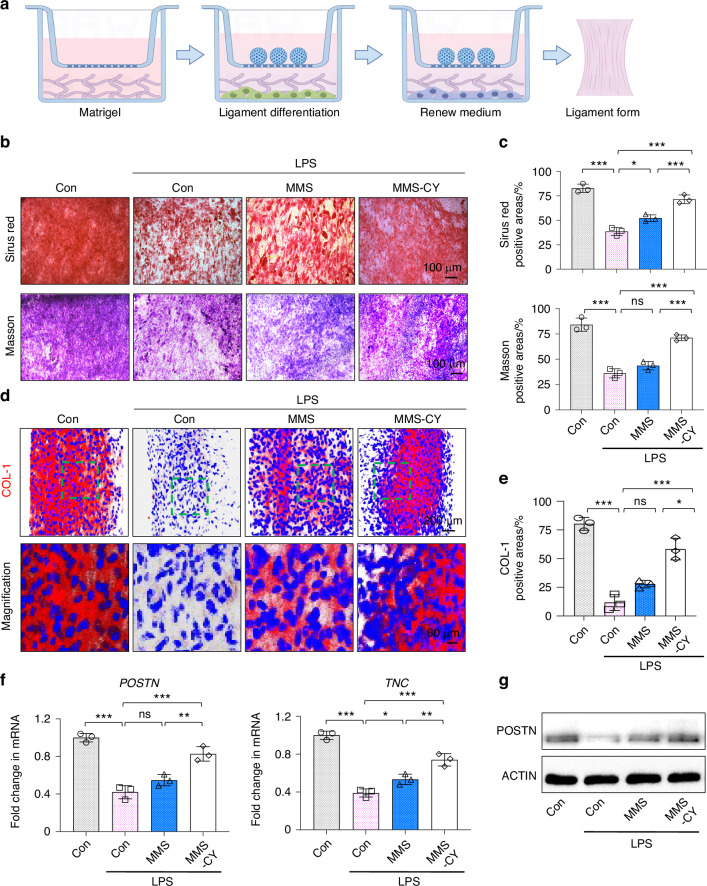
MMS-CY rescues the impaired ligament-forming capacity of PDLSCs under the inflammation stimulus. **a** Schematic diagram of MMS-CY restoring impaired ligament-forming capacity of PDLSCs under the inflammatory condition. **b** Sirius red and Masson’s trichrome staining of PDLSCs after the coculture with MMS and MMS-CY for 14 days respectively. **c** Quantification of Sirius red positive areas and Masson positive areas in total areas on day 14 (*n* = 3 biologically independent samples, by one-way ANOVA Turkey’s multiple comparison test: ns: not significant, ***P < 0.001, **P < 0.01, *P < 0.05). **d** Immunofluorescence staining of COL-1 of MMS and MMS-CY-treated PDLSCs under the inflammatory condition. **e** Semi-quantification of COL-1 (*n* = 3 biologically independent samples, by one-way ANOVA Turkey’s multiple comparison test: ns: not significant, ***P < 0.001, *P < 0.05). **f** RT-qPCR of the gene exression of POSTN and TNC (*n* = 3 biologically independent samples, by one-way ANOVA Turkey’s multiple comparison test: ns: not significant, ***P < 0.001, **P < 0.01, *P < 0.05). **g** Western blot of POSTN in MMS and MMS-CY-treated PDLSCs under the inflammatory condition. Data are represented as mean ± SD

The pathological features of periodontitis are characterized by the progressive bone loss and the destruction of periodontal fibers. Recovering the structure of periodontal fibers is essential for transmitting the occlusion force and maintaining the healthy periodontal bone turnover.^22^ Therefore, we spread a layer of matrigel on the lower chamber to provide matrix support for PDLSCs (Fig. 4a). To induce the periodontium-like collagen production, we added the arsobic acid into the medium to stimulate the collagen secretion. Then, we added the MMS-CY into the upper chamber. Sirius red and Masson trichrome staining demonstrated that the parallel periodontium-like collagen formed in control groups without the LPS treatment. By contrast, the persistent inflammatory stimulus significantly reduced the percentage of collagen deposition areas. Supplementation with MMS-CY during the ligament induction recovered the ability to form a parallel collagen matrix (Fig. 4b, c). Collagen I is the primary matrix component of the periodontium.^23^ Therefore, we examined the expression of collagen I after the ligament differentiation induction. Immunofluorescence staining showed that the application of MMS-CY recovered collagen I expression, which also presents a parallel-structure distribution (Fig. 4d, e). Tenascin-C (Tnc) and periostin are two representative markers for the periodontal ligament.^24–26^ RT-qPCR results also showed that the inflammation stimulus suppressed mRNA levels of tenascin-C and periostin, which was reversed by the supplementation of MMS-CY (Fig. 4f). Moreover, western blot results presented the same trend (Fig. 4g). These results demonstrated that MMS-CY could act as “a drug island” to restore the collagen fiber-forming ability of inflammatory PDLSCs.

### MMS-CY restored the impaired status of inflammatory PDLSCs by reducing the DNA injury

**Fig. 5:**
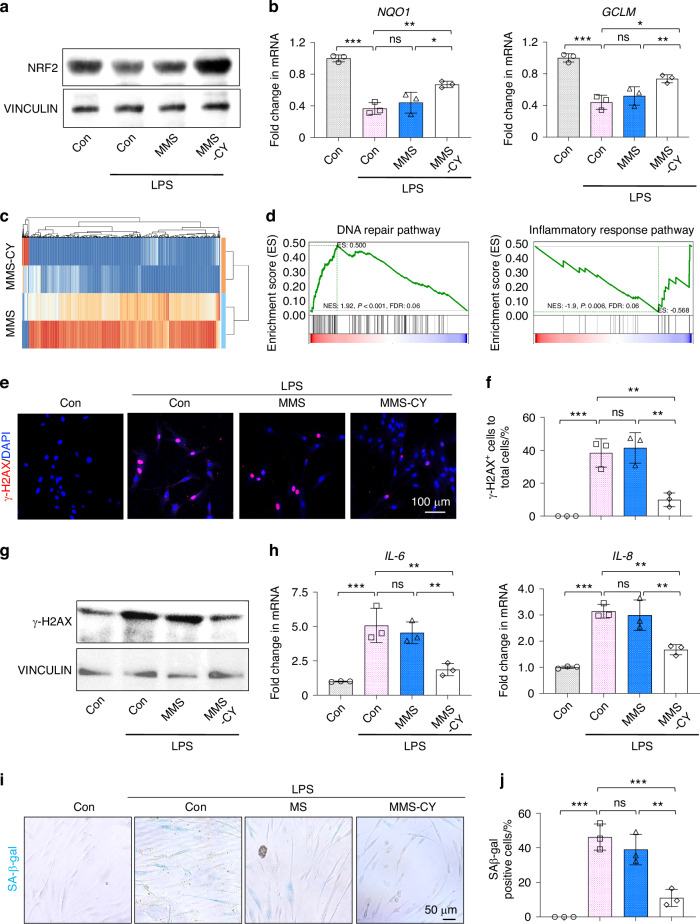
MMS-CY restores the impaired function of PDLSCs by reducing the DNA injury. **a** Western blot of NRF2 in MMS and MMS-CY-treated PDLSCs under the inflammatory condition. **b** RT-qPCR of the gene expression of NQO1 and GCLM. (*n* = 3 biologically independent samples, by one-way ANOVA Turkey’s multiple comparison test: ns: not significant, ***P < 0.001, **P < 0.01, *P < 0.05). **c** Significantly upregulated and downregulated genes (MMS vs MMS-CY). **d** GSEA shows a significant increase in DNA repair gene signatures and a significant decrease in inflammatory response pathway gene signatures in MMS-CY groups. **e** Immunofluorescence staining of γ-H2AX of MMS and MMS-CY-treated PDLSCs under the inflammatory condition. **f** Semi-quantification of γ-H2AX (*n* = 3 biologically independent samples, by one-way ANOVA Turkey’s multiple comparison test: ns: not significant, ***P < 0.001, **P < 0.01). **g** Western blot of γ-H2AX in MMS and MMS-CY-treated PDLSCs under the inflammatory condition. **h** RT-qPCR of the gene expression of IL-6 and IL-8 (*n* = 3 biologically independent samples, by one-way ANOVA Turkey’s multiple comparison test: ns: not significant, ***P < 0.001, **P < 0.01). **i** SAβ-gal staining of MMS and MMS-CY-treated PDLSCs. Blue cells are senescent cells. **j** Semi-quantification of SAβ-gal (*n* = 3 biologically independent samples, by one-way ANOVA Turkey’s multiple comparison test: ns: not significant, ***P < 0.001, **P < 0.01). Data are represented as mean ± SD

Having established that MMS-CY could restore the osteogenic capacity and ligament-forming capacity of inflammatory PDLSCs, we next aim to elucidate the underlying molecular mechanisms. NRF2 is a critical therapeutic target that can be efficiently activated by the cordycepin and associated with periodontal bone regeneration.^27^ Western blotting results showed that MMS-CY increased the NRF2 protein expression (Fig. 5a). RT-qPCR results also showed that MMS-CY remarkably increased the mRNA expression of antioxidant genes NQO1 and GCLM, compared with other groups (Fig. 5b). Therefore, the RNA-seq was carried out for the comparison between the MMS and MMS-CY groups. A total of 889 genes were identified as differentially expressed genes (858 up and 31 down) (Fig. 5c). Then, the GSEA analysis found that treatment with MMS-CY increased the expression level of Cell cycle and DNA repair signature genes in PDLSCs. Interestingly, the GSEA analysis also showed that treatment with MMS-CY reduced the expression level of inflammatory response pathway signature genes (Figs. 5d and S2).

Next, the DNA injury-related protein γ-H2AX was examined by immunofluorescence staining. The MMS-CY group displayed a decreasing percentage of γ-H2AX positive cells compared with other groups (Fig. 5e, f). Moreover, western blot results display the same trend (Fig. 5g). In addition, PDLSCs treated with the LPS solution increased the expression of senescence-related genes, including IL-8 and IL-6 (Fig. 5h). Previous studies reported that persistent inflammatory stimulus could result in the premature senescence of stem cells, in which DNA injury is one of the typical symptoms.^28,29^ Next, we investigated the influence of MMS-CY on the PDLSCs’ senescence. Senescence-associated β-galactosidase (SA-β-gal) is regarded as a marker of cell senescence.^30^ Results showed that persistent treatment of PDLSCs with LPS enhanced SA-β-gal activity, suggesting that LPS realized the successful induction of premature cell senescence. Interestingly, the supplementation with MMS-CY remarkably decreased the percentage of SA-β-gal^+^ PDLSCs (Fig. 5i, j). Overall, our results demonstrated that MMS-CY was able to inhibit PDLSC senescence to restore their impaired function under inflammatory condition.

### MMS-CY inhibited osteoclast activity

**Fig. 6:**
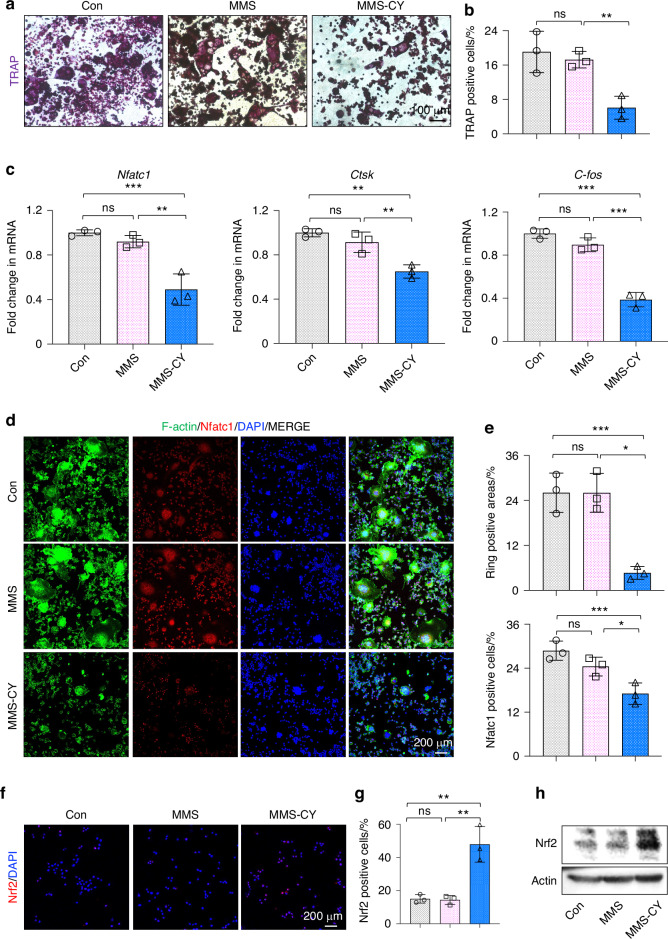
MMS-CY inhibits osteoclast differentiation. **a** TRAP staining of RAW264.7 after coculture with MMS and MMS-CY. **b** Quantification of TRAP-positive cells. (*n* = 3 biologically independent samples, by one-way ANOVA Turkey’s multiple comparison test: ns: not significant, ns: not significant, **P < 0.01). **c** RT-qPCR of the osteoclastic gene expression *Nfatc*, *Ctsk*, and *C-fos*. (*n* = 3 biologically independent samples, by one-way ANOVA Turkey’s multiple comparison test: ns: not significant, ***P < 0.001, **P < 0.01). **d** Immunofluorescence staining of F**-**actin and Nfatc1 of MMS and MMS-CY-treated RAW264.7. **e** Semi-quantification of ring positive areas and Nfatc1 positive cells (*n* = 3 biologically independent samples, by one-way ANOVA Turkey’s multiple comparison test: ns: not significant, ***P < 0.001, *P < 0.05). **f** Immunofluorescence staining of Nrf2 of MMS and MMS-CY-treated RAW264.7. **g** Semi-quantification of Nrf2 positive cells (*n* = 3 biologically independent samples, by one-way ANOVA Turkey’s multiple comparison test: ns: not significant, **P < 0.01). **h** Western blot of Nrf2 in MMS and MMS-CY-treated RAW264.7. Data are represented as mean ± SD

Periodontal bone metabolism under the inflammatory condition is characterized by the excessive osteoclast activity and attenuated osteoblast activity.^31^ Therefore, we also examined the osteoclast activity. First, the RANKL stimulation was given to RAW264.7 cells to induce bone-resorbing multinucleated osteoclasts. The positive areas for tartrate-resistant acid phosphatase (TRAP) staining in the MMS-CY groups were lowest compared with other groups (Fig. 6a, b). Compared with control and MMS groups, the supplementation with MMS-CY significantly inhibited the expression of osteoclastic genes, including the nuclear factor of activated T cells (Nfatc), cathepsin K (Ctsk), and C-fos (Fig. 6c). Together, above results indicated that MMS-CY upregulated the number of the osteoclasts and promoted the osteoclastic gene expression. Furthermore, immunofluorescence staining showed that the osteoclasts in control and MMS groups displayed a large size and ring-like F-actin sealing zones, accompanied by a higher percentage of Nfatc-positive cells. Remarkably, the application of MMS-CY reduced the number of F-actin sealing zone areas and decreased the percentage of Nfatc1-positive cells (Fig. 6d, e). It has been demonstrated that the activation of NRF2 could efficiently inhibit the osteoclast activity. Interestingly, we found that the application of MMS-CY efficiently elevated the protein expression of Nrf2 in Raw 264.7, as evidenced by the immunofluorescence staining and western blotting results (Fig. 6f–h). Altogether, these results suggested that MMS-CY possesses an excellent inhibitory effect on the osteoclast activity.

### MMS-CY supplementation promotes periodontal bone regeneration

**Fig. 7:**
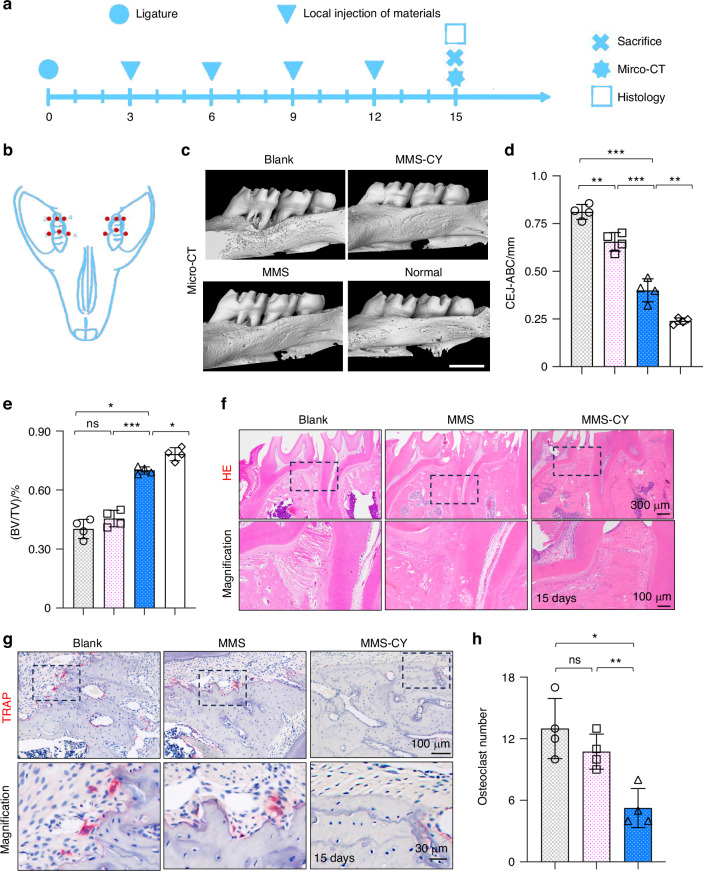
MMS-CY inhibits the bone loss in rat ligature-induced periodontitis. **a** Experiment schedule of in vivo periodontitis study. **b** Schematic diagram of the establishment of rat periodontitis experiment. **c** 3D reconstructed digitized images of the maxillary first and second molars analyzed by micro-CT. **d** Quantitative analysis of BV/TV. (*n* = 4 biologically independent samples, by one-way ANOVA Turkey’s multiple comparison test: ***P < 0.001, **P < 0.01). **e** Quantitative analysis of CEJ-ABC. (*n* = 4 biologically independent samples, by one-way ANOVA Turkey’s multiple comparison test: ns: not significant, ***P < 0.001, *P < 0.05). **f** H&E staining images in different groups. **g** TRAP staining images in different groups. **h** Quantitative analysis of the osteoclast number in periodontal tissues. (*n* = 4 biologically independent samples, by one-way ANOVA Turkey’s multiple comparison test: ns: not significant, **P < 0.01, *P < 0.05). Data are represented as mean ± SD

**Fig. 8:**
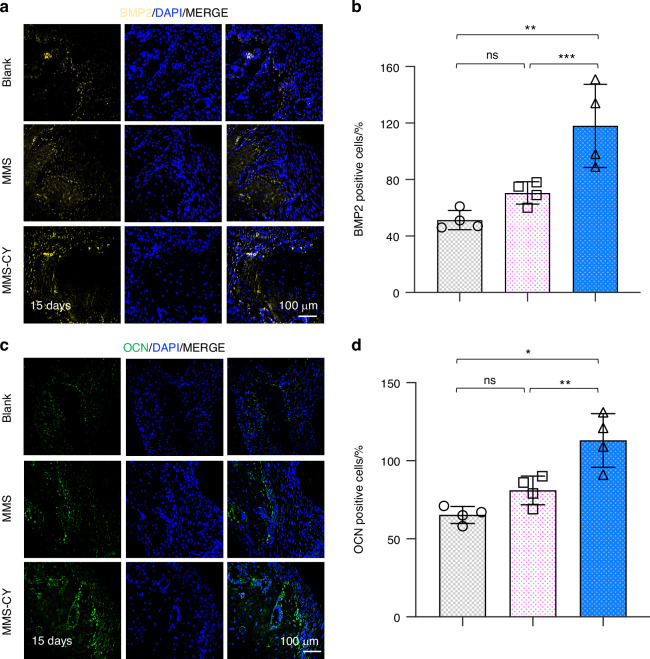
MMS-CY promotes the bone formation in rat ligature-induced periodontitis. **a** Immunofluorescence staining of staining of BMP2 in periodontal tissues on day 15 after the MMS-CY administration. **b** Quantitative analysis of the number of BMP2 positive cells in the periodontal tissues. (*n* = 4 biologically independent samples, by one-way ANOVA Turkey’s multiple comparison test: ns: not significant, ***P < 0.001, **P < 0.01). **c** Immunofluorescence staining of staining of OCN in periodontal tissues on day 15 after the MMS-CY administration. **d** Quantitative analysis of the number of OCN positive cells in the periodontal tissues. (*n* = 4 biologically independent samples, by one-way ANOVA Turkey’s multiple comparison test: ns: not significant, **P < 0.01, *P < 0.05). Data are represented as mean ± SD

Given the positive effects of MMS-CY on the osteogenesis and ligament-forming capacity of PDLSCs, we performed in vivo animal studies to further demonstrate their therapeutic function of MMS-CY on the periodontal bone loss. As shown in the schematical diagram (Fig. 7a, b), we successfully established a ligature-induced rat periodontitis model at day 0 and injected different kinds of microspheres into the gingival sites every three days. After 15 days, the micro-CT analysis was performed to reconstruct 3D images and to calculate parameters that are related to periodontal bone regeneration, including the BV/TV (Bone volume/tissue volume, %) and CEJ-ABC (the Cemento-enamel junction and the alveolar bone crest). Micro-CT results demonstrated that the ligation method induced the periodontitis in the rats, causing the remarkable bone loss and decreased bone height around the molar (the blank group). However, application of MMS was unable to reduce the periodontal bone loss effectively. In contrast, the injection with MMS-CY remarkably inhibited the bone resorption, which was reflected by the higher BV/TV and decreased the CEJ-ABC (Fig. 7c–e). Hematoxylin-eosin (HE) showed that the periodontal tissue displayed a denser and more organized collagen fibers and higher volume of alveolar bone in the MMS-CY groups. The periodontal tissue in the blank and MMS groups displayed a disorganized and sparse arrangement of fibers and decreased volume of alveolar bone (Fig. 7f). Furthermore, the results from the TRAP staining revealed that MMS-CY reduced the number of osteoclasts along the bone margin, while the number from the blank or MMS groups displayed no significant difference (Fig. 7g, h). Furthermore, the immunofluorescence staining showed that the expression level of osteogenic proteins, including BMP2 and OCN, declined in blank and MMS groups. Interestingly, their expression significantly elevated after the injection with MMS-CY (Fig. 8a–d). Above results indicated that the application of MMS-CY effectively alleviates the rat experimental periodontitis and delays the progression of the periodontal bone loss.

## Discussion

The disequilibrium of the excessive osteoclast activity and impaired osteogenesis are critical factors that accompany the persistent progression of the bone loss and hinder the healing of bone defects in periodontitis condition.^32^ Moreover, during the process of periodontitis, the persistent bone loss could cause the degradation of the periodontal ligament fiber, which in turn could aggravate the bone loss. Therefore, on the other hand, maintaining or recovering the structure of periodontal ligament fiber also exerts a crucial role in promoting periodontal bone repair and regeneration.^33^ The current treatments for periodontitis are incapable of regenerating the alveolar bone in periodontitis efficiently. In our study, we have created adhesive and mineralized hydrogel microspheres loaded with the traditional compound cordycepin (MMS-CY), which displayed the enhanced alveolar bone regeneration by improving the osteogenesis and inhibiting the osteoclastic activity in periodontitis rats.

The periodontium tissue consists of the gingiva, periodontal ligament and alveolar bone, which forms into an integral tissue structure. It is relatively difficult for ultraviolet light to completely penetrate the gingiva to realize the crosslinking process of hydrogel in alveolar bone defect.^16^ Moreover, the method by injecting therapeutic drugs is unable to realize the retainment of drugs in the diseased area, which results into the difficulty in maintaining the therapeutic concentration in vivo.^34^ Therefore, the adhesive ability and porous structure of carrier hydrogel are needed to load drugs and maintain their continuous release. Hydrogel microspheres have been regarded as advanced drug delivery tools with a wide range of particle sizes. Minimally invasive operations can be applied to realize the local injection of microspheres. Different kinds of microspheres can be constructed by various kinds of technologies, including microfluidics, batch emulsion, electro-hydrodynamic spraying, and photolithography techniques.^35^ Monodisperse microspheres with about 100–200 μm diameter can be produced by the batch emulsion method.^36^ To improve the adhesive property of microspheres, mussel-inspired adhesion method have been attempted in many studies. By means of the modification with the polydopamine (PDA) solution, the adhesive capacity of biological materials can be significantly improved.^37,38^ Therefore, the similar technology may be used to increase the adhesive ability of hydrogel microspheres on the alveolar bone.

Therefore, to overcome the limitations of periodontium anatomy, the adhesive and injectable hydrogel microspheres were constructed to realize the adhesion to the alveolar bone and the directional drug delivery. Intending to promote the bone formation, osteoinductive inorganic elements such as calcium phosphates are usually introduced. The PDA coating could increase more nucleation sites that could be used for apatite deposition, eventually forming biomineralization layers. In our study, hydrogel microspheres produced by batch emulsion method were modified with PDA and biomineralized by simulated body fluids, which formed injectable and mineralized microsphere that was suitable for the alveolar bone. This delivery system could adhere to the wet surface of alveolar bone and possess controlled drug releasing property. In vitro experiments found that MMS-CY could promote the PDLSCs’ migratory capacity. It may be attributed to their biomineralization layer that MMS-CY is able to promote the PDLSCs’ osteogenic capacity. Moreover, without the osteogenic induction, MMS-CY is also conducive to the formation of parallel-collagen structure and inhibits their excessive osteoclast activity.

Cordycepin (3′-deoxyadenosine) is regarded as the derivative of the nucleoside adenosine that displays huge potential in biomedical areas. Many studies have demonstrated that it possessed some therapeutic properties, including anti-inflammation, anti-aging, and anti-tumor.^10^ The above evidence suggests that cordycepin has enormous prospect in clinical application. Recently, many studies have attempted their utilization in the osteoporosis by inhibiting the osteoclast activity, which indicated that it held immense potential in treating the periodontitis.^12^ However, high concentrations of cordycepin inhibit the cell cycle and result into the liver and kidney toxicity. Therefore, taking advantage of natural nanoparticles loaded with cordycepin may be potential methods for realizing the controlled release and reduce these adverse reactions.^13^ In our study, the combination of cordycepin-loaded nanoparticles and porous microspheres realized the controlled drug releasing, which may be an important reason for the excellent periodontal bone regeneration effect in vivo.

Some anti-bacterial and anti-inflammatory drugs have displayed some potential in delaying the progression of periodontal bone loss to some extent.^39^ However, these methods are incapable of restoring the impaired function of periodontal tissue and thoroughly motivating the endogenous regenerative potential. Previous studies have demonstrated that PDLSCs isolated from patients with periodontitis displayed the reduced osteogenic capacity compared with normal PDLSCs from healthy individuals. The reduced osteogenic capacity of PDLSCs largely contributes to the impaired potential in alveolar bone repair.^40^ One of the typical symptoms during the progress of senescence is the chronic inflammatory stimulus, which is a major hurdle for the tissue healing.^41,42^ Recently, NRF2 has been linked to the premature senescence of stem/progenitor cells and the dysfunction in humans and mice.^43^ Here, in addition to the pro-osteogenic effect of MMS-CY on PDLSCs, we showed that MMS-CY significantly activated the NRF2 pathway. Furthermore, the treatment with MMS-CY remarkably suppresses the expression levels of inflammation-related genes and the amount of SA-β-gal positive cells. Senescent cells often displayed the reduced proliferative activity and the increased DNA injury.^44^ In consistency with it, our study found that MMS-CY efficiently recovers the proliferative capacity of PDLSCs under the inflammation condition and reduces their DNA injury to some extent, which may explain the enhanced migratory and osteogenic capacity in PDLSCs from MMS-CY-treated groups.

## Methods

### Cells isolation and culture

The RAW 264.7 cells were brought from the American Type Culture Collection (ATCC, Manassas, USA). The PDLSCs were collected from the periodontal ligament in healthy individuals. The experimental procedures were approved by the Ethics Committee of Peking University School of Stomatology, Beijing, China (PKUSSIRB-202385020). In general, the premolars from patients who received orthodontic treatment and aged 12–15 years were gathered. Cells were seeded in *α*-modified eagle’s medium (*α*-MEM) (Hyclone) supplemented with 10% fetal bovine serum (FBS) (Thermo Fisher Scientific) and 1% penicillin/streptomycin at 37 °C_._ The passage 3–5 PDLSCs were prepared for further experiments. We chose the 5 μg/mL LPS as the experimental concentration.

### Animals

All experimental operations were approved by the Animal Use and Care Committee of Peking University (DLASBD0284). A ligature-induced rat periodontitis animal model was used to evaluate the in vivo therapeutic effects of MMS or MMS-CY on the ligature-induced bone loss. Generally, twenty male SD rats (6–8 week-old) were kept for 1-week adaptive time and then divided into four groups randomly. Firstly, all rats were anesthetized by injecting the sodium pentobarbital intraperitoneally. Then, a 3-0 ligature wire was fixed around the maxillary first molars. According to the drug release experiments, we eventually established the amount of immobilized CY on microspheres is about 30 μg/mg. Therefore, we injected 2 mg of MMS-CY with the volume of 200 μL via periodontal administration in vivo. We chose the proximal, middle, and distal sites of palatal gingiva as the injection location and maintained for every 3 days. After 15 days, overdose anesthesia was used to execute rats.

### Micro-CT

The maxillas were examined by means of micro-CT (Belgium) to evaluate the condition of alveolar bone. CT Vox and Data Viewer software were used to reconstruct three-dimensional (3D) images. BV/TV and the vertical distance from the cementoenamel junction (CEJ) to alveolar bone crest (ABC) (CEJ-ABC) in different groups were also analyzed.

### Drug-loading PLGA

Drug-loading nanoparticles were obtained according to our previous study.^42^ The ratio of drugs to the polymer weight with 5% (w/w) was established to construct the polymer solution.

### Quantitative real-time polymerase chain reaction (qRT-PCR)

1 mL Trizol solution (Thermo Fisher Scientific) was added into the cells. Total RNA was extracted according to the protocol. A 20 μL system was transcribed to get cDNA samples. qPCR was carried out with the TB green premix Ex Taq kit (Takara) and performed in a 900HT Fast Time PCR. All sequences of the qPCR primers were provided in Table S3 Supporting Materials (Shanghai Sangon Biotechnology, Shanghai, China).

### Western blot analysis

The proteins were gathered by adding RIPA solution (Beyotime Biotechnology, China) with the supplementation of suitable protease inhibitors (Beyotime Biotechnology, China). The BCA method was adopted to determine the concentration. The proteins were heated in a 99 °C condition for 8 min and then keep at −80 °C for further experiments. Proteins were run on 10% sodium dodecyl sulfate-polyacrylamide for 90 min at 120 V. Then, the protein was transferred to the nitrocellulose membrane for 60–90 min at 100 V voltage (Millipore). Transferred membranes were sealed in 5% BSA solution (Solarbio). Diluted primary antibodies were incubated with the membranes. Secondary antibodies with a concentration of 1:5 000 dilution were incubated. Finally, the bands were recorded. (Thermo Fisher Scientific).

### Immunofluorescence staining

Briefly, PDLSCs were harvested and fixed and then washed with PBS thrice. Then, the samples were placed in 0.1% Triton solution (Beyotime Biotechnology, China) for 5 min. Next, 1% BSA solution was used to seal the samples for 30 min. Diluted primary antibody was applied into the samples overnight at 4 °C. Corresponding secondary antibodies (Thermo Fisher Scientific) were applied for 1.5 h. Finally, DAPI was used to stain the cell nuclei. CLSM (LSM 510, Zeiss, Germany) was photographed.

### Alkaline phosphatase staining and activity

After the osteogenic induction for 7 days, the cells were fixed and then stained with staining solution. The staining results were observed and photographed by the microscope. To examine ALP activity, ALP activity detection kit (Beyotime Biotechnology, China) was applied.

### Alizarin red staining

After the osteogenic differentiation induction for 14 days, the cells were examined by ARS staining to observe mineralized deposition. Generally, the differentiated cells were fixed. ARS solution was applied in the culture (Beyotime Biotechnology, China). Finally, the cells were photographed.

### β-galactosidase (SA-β-gal)

1 × 10^4^ PDLSCs were cultured in the 24-well plate. After 10-day LPS stimulation and subsequent treatment with microspheres, the PDLSCs were fixed. Then, the SA-β-gal staining was performed according to the protocol (Cell Signaling Technology).

### Transwell assay

2 × 10^4^ PDLSCs were cultured in the transwell. MMS and MMS-CY were supplemented into the lower chambers. To minimize the effects of serum, serum-free medium was applied. Finally, the migrated PDLSCs were stained with crystal violet and photographed and counted by microscopy.

### TRAP staining of RAW264.7 cells

1 × 10^5^ RAW264.7 cells were incubated with osteoclast induction medium. After the continous 7-day culture, the differentiated cells were stained according to the protocol (Sigma-Aldrich). Cells with dark red staining and three or more cell nuclei were defined as osteoclasts.

### RNA-seq

RNA isolation, library construction, and subsequent sequencing were carried out according to the previous protocol.^42^ Transcripts and genes with the fold change (>1.5 or <0.66) and *P*-value < 0.05 were screened out.

### Histological analysis

The harvested maxillas were fixed in 4% PFA solution overnight and then decalcified in EDTA solution, and finally embedded in paraffin. Tissue sections were acquired at 8 µm thickness. H&E and Masson’s trichrome staining were carried out to assess the regenerated alveolar bone. Moreover, the TRAP staining was carried out to calculate the number of osteoclasts. The number of OCN and BMP2 positive cells was examined by the immunofluorescence staining.

### Synthesis of MMS-CY

The PDA-modified microspheres were prepared according to our previous study.^45^ Then, PDA-modified microspheres were then immersed in 5-times SBF (5SBF) and CaCl_2_ solution at 37 °C for 12 h to finish the process of apatite deposition. Finally, the immersion adsorption method was used to load the PLGA nanoparticles on the microspheres.

### Characterization of MMS-CY

1) The microstructure of freeze-dried microsphere was examined by SEM; 2) TEM was used to examine the structure of PLGA nanoparticles. 3) Dil (Solarbio, China) was used to stain the nanoparticles, and the labeled nanoparticles were examined by fluorescence microscope.

### Drug release

MMSs were mixed with CY-loaded PLGA nanoparticles and kept for dynamic shaking at 37 °C for 24 h. Then, the mixed MMS was washed with PBS solution to get rid of the unabsorbed nanoparticles. MMS-CY was dialyzed in 30 mL deionized water at 37 °C on a thermostatic oscillator. At different set time points, 1 mL sample was gathered, while 1 mL deionized water was then supplemented. The drug concentration was examined by UV/Visible Photometer-5100.

### Swelling experiments

1.5 mL EP tube with 1 mL deionized water and 3 mg microspherese were kept on a thermostatic oscillator at 37 °C. The samples were gathered at different time points. After removing the supernatant, the weight of different kinds of microspheres was recorded. The calculation formula is :(wet weight-dry weight)/dry weight.

### Osteogenic differentiation induction

50 000 PDLSCs were maintained in 12-well plates. The osteogenic differentiation medium is composed of the growth medium that contained 10 nmol/L dexamethasone, 5 mmol/L β-glycerophosphate, and 0.05 mmol/L l-ascorbic acid 2-phosphate. The PDLSCs were induced to osteogenic differentiation for 7 days or 14 days and then used for the ALP and ARS staining.

### Ligament differentiation induction

The medium for the ligament differentiation is composed of the growth medium that contained 10 ng/mL TGF-β1, 0.05 mmol/L l-ascorbic acid 2-phosphate. The PDLSCs were induced to ligament differentiation for 14 days and then used for the sirius red or masson trichrome staining.

### Biocompatibility of microspheres

To evaluate the biocompatibility of MMS and MMS-CY, living/dead assay was adopted (Solarbio, China). Briefly, PDLSCs were cultured in a 24-well plate with microspheres. On day 3, the live/dead cell staining reagents was added into the culture according to the protocol. CCK-8 experiment (Beyotime Biotechnology, China) was further conducted to detect cell cytotoxicity. Briefly, PDLSCs (0.5 × 10^4^ /mL) were cultured with microspheres. 10 μL reaction solution was collected on day 1, 2, and 3. The absorbance was recorded by use of a microplate reader.

### Cell migration assay

The upper chamber contained different kinds of microspheres. PDLSCs were cultured in the lower chamber (Corning). A 200 μL pipette tip was used to create a straight scratch. To minimize the effects of serum, a serum-free medium is adopted. The healing condition was examined and measured by the Image J software.

### Scanning electron microscopy

In general, different kinds of microspheres were frozen at −40 °C for about 6 h. Then, these microspheres were dried in a vacuum freeze dryer overnight. The specimens were processed by a gold sputter coating and observed by SEM (Hitachi SU8020, Japan).

### Statistical analysis

All data were represented as the mean ± SD. Statistical significance was analyzed by unpaired two-tailed t-tests (for two-group comparison) or one-way analysis of variance with Tukey’s post hoc test (for multiple-group comparisons). Statistical significance was accepted at *P* < 0.05.

## Supplementary information

SUPPLEMENTAL MATERIAL

## Data Availability

All datas are provided in the paper. The original data of RNA-seq in the paper has been uploaded into NCBI database with the identifier GSE276691.
