# Supplementary material for: Adhesive and injectable hydrogel microspheres for NRF2-mediated periodontal bone regeneration
Source: Int J Oral Sci. 2025 Jan 10;17:7. doi: 10.1038/s41368-024-00340-w (PMC11717957; doi:10.1038/s41368-024-00340-w)
Supplement: Supplementary file 1 — SUPPLEMENTAL MATERIAL [file 41368_2024_340_MOESM1_ESM.pdf]

Supporting Information

Supplementary Figures

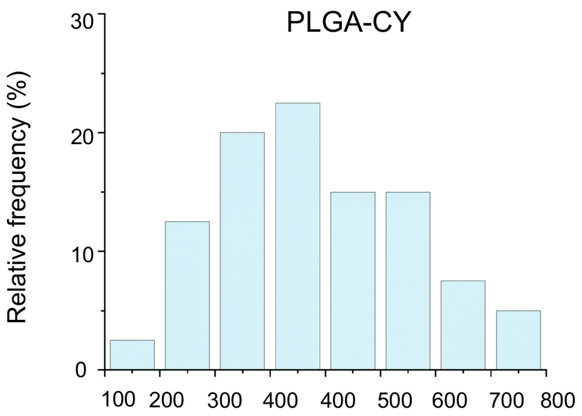

Supplementary Figure 1. Size distribution of MMS-CY that are determined by the dynamic light scattering.

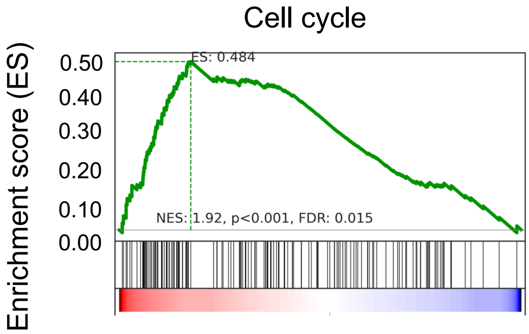

Supplementary Figure 2. GSEA shows a significant increase in cell cycle gene signatures and a significant decrease in inflammatory response pathway gene signatures in MMS-CY groups.

Supplementary Table 1. List of reagents or resources used in the study.

REAGENT or RESOURCE	SOURCE	IDENTIFIER
**Antibodies**		
Rabbit polyclonal anti-γ-H2AX	Abcam	Cat#AB11175
Rabbit monoclonal anti-Bmp2	Abcam	Cat#AB214821
Rabbit monoclonal anti-Periostin	Abcam	Cat#AB315104
Mouse monoclonal anti-Actin	ZSGB-BIO	Cat#TA-09
Mouse monoclonal anti-Vinculin	Proteintech	Cat#66305-1-lg
Mouse monoclonal anti-GADPH	Proteintech	Cat#60004-1-lg
Rabbit polyclonal anti-NRF2	Proteintech	Cat#16396-1-AP
Rabbit polyclonal anti-Col1	Abcam	Cat#AB138492
Rabbit polyclonal anti-NFATc1	Santa Cruz Biotechnology	Cat#SC-13033
Rabbit polyclonal anti-OCN	Abcam	CAT#AB93876
Goat anti-Mouse IgG(H+L) Cross-Adsorbed Secondary Antibody Alexa Fluor 488	Thermo Fisher Scientific	Cat#A-11001
Goat anti-Rabbit IgG(H+L) Cross-Adsorbed Secondary Antibody Alexa Fluor 488	Thermo Fisher Scientific	Cat#A-11008
Goat anti-Rabbit IgG(H+L) Cross-Adsorbed Secondary Antibody Alexa Fluor 594	Thermo Fisher Scientific	Cat#A32740
Donkey anti-Mouse IgG(H+L) Highly Cross-Adsorbed Secondary Antibody, Alexa Fluor Plus594	Thermo Fisher Scientific	Cat#A32744

Supplementary Table 2. List of chemicals and recombinant proteins used in the study.

**Chemicals, peptides, and recombinant proteins**		
α-MEM	Thermo Fisher Scientific	Cat# 12571063
DMEM	Thermo Fisher Scientific	Cat# 11885084
Penicillin-Streptomycin	Hyclone	Cat#15070063
Trypsin-EDTA	Hyclone	Cat#SH30042.01
TRIzol Reagent	Thermo Fisher Scientific	Cat#15596026
Collagenase Type I	Thermo Fisher Scientific	Cat#17100017
Crystal Violet Stain solution	Solarbio	Cat#G1062
RNeasy mini Kit	Qiagen	Cat#74104
4',6-diamidino-2-phenylindole	Beyotime Biotechnology	Cat#C1002
Dispase	Roche	Cat#10269638001
Matrigel	Thermo Fisher Scientific	Cat#A1413302
RIPA Buffer	Thermo Fisher Scientific	Cat#89900
L-Glutamine	Thermo Fisher Scientific	Cat#25030081
L-Ascorbic acid	Sigma-Aldrich	Cat#A5960
SYBR Green Supermix	Thermo Fisher Scientific	Cat#4385612
Fetal bovine serum（FBS）	Thermo Fisher Scientific	Cat#10099-141
Dexamethasone	Sigma-Aldrich	Cat#D8893
β-glycerophosphate	APEXBIO	Cat#C4347
DiR	Solarbio	Cat#HY-D1048
Cordycepin	Selleck	Cat#S3610
PVA solution	Sigma-Aldrich	Cat#341584
Tris-HCl solution	Solarbio	Cat#T1010
Polydopamine	Sigma-Aldrich	Cat#62-31-7
TB green premix Ex Taq kit	Takara	Cat#RR42WR
TBS	Solarbio	Cat#T1080
TGF-β1	Peprotech	Cat#100-21
b-Glycerophosphate	APEXBIO	Cat#3408-09-8
L-Ascorbic acid	Sigma-Aldrich	Cat#A5960
Triton-X	Beyotime Biotechnology	Cat#ST1723
Tween	Sigma-Aldrich	Cat#P9416
**Critical commercial assays**		
ReverTra Ace qPCR RT Kit	TOYOBO	Cat#FSQ-101
Alizarin red S stain Kit	Beyotime Biotechnology	Cat#G1262
Senescence β-Galactosidase Staining Kit	Cell Signaling Technology	Cat#9860
H&E staining Kit	Solarbio	Cat#G1120
Masson's trichrome Kit	Solarbio	Cat#G1340
Alp staing kit	Beyotime Biotechnology	Cat#G1340
Pierce BCA protein assay Kit	Thermo Fisher Scientific	Cat#23225
Enhanced Chemiluminescence Western Blotting Detection Kit	Thermo Fisher Scientific	Cat#34577
Live/Dead assay Kit	Solarbio	Cat#CA1630
CCK-8 aasay kit	Beyotime Biotechnology	Cat#

Supplementary Table 3. List of primers used in the study.

Gene	Assay	Forward (5'-3')	Reverse (5'-3')
hGAPDH	qRT-PCR	GTTCCAGTATGACTCTACCCACG	CATTTGATGTTAGCGGGATCTCG
mNFATc1	qRT-PCR	AACGCCCTGACCACCGATAGCACT	CCCGGCTGCCTTCCGTCTCATA
mCtsk	qRT-PCR	CTTCCAATACGTGCAGCAGA	TCTTCAGGGCTTTCTCGTTC
mGadph	qRT-PCR	AATGGTGAAGGTCGGTGTG	GAAGATGGTGATGGGCTTCC
mC-fos	qRT-PCR	CCAGTCAAGAGCATCAGCAA	AAGTAGTGCAGCCCGGAGTA
hIL-6	qRT-PCR	GAAGATTCCAAAGATGTAGCCGC	TTACATGTCTCCTTTCTCAGGGC
hIL-8	qRT-PCR	GAGGGAGAGAAGCAACTACAGAC	GTGGGTCAGTATGTGAGAGGAAG
hRUNX2	qRT-PCR	ACTCACCTCTTCAGAACGAATTG	CCATCTTTGGAAGGTTCAGGTTG
hOSX	qRT-PCR	GGATGCCAGGAAAGGTTCTG	CCAGGTGTGGAGTTCCTGATGT
hBGALP	qRT-PCR	AAATAGCCCTGGCAGATTCC	CAGCCTCCAGCACTGTTTAT
hGCLM	qRT-PCR	TGTCTTGGAATGCACTGTATCTC	CCCAGTAAGGCTGTAAATGCTC
hNQO1	qRT-PCR	CAGCTCACCGAGAGCCTAGT	GAGTGAGCCAGTACGATCAGTG
hPeriostin	qRT-PCR	AATGCTGCCCTGGCTATATG	GCATGACCCTTTTCCTTCAA
hTenascin-C	qRT-PCR	ACGAGGGTGGTCTGGAAATG	GGATGGCAAATACACGGATAAAG

Supplementary Table 4. Software and Algorithms.

Gene ontology	https://geneontology.org/
GSEA 3.0	http://software.broadinstitute.org/gsea/index.jsp
µCT Evaluation CTAn software	https://www.blut-scientific.com/bruker-micro-ct-software/
Graph Pad Prism 9.0	https://www.graphpad.com/
Image Pro Plus 6.0 software	http://fiji.sc
